# A novel quantitative high-throughput screen identifies drugs that both activate SUMO conjugation via the inhibition of microRNAs 182 and 183 and facilitate neuroprotection in a model of oxygen and glucose deprivation

**DOI:** 10.1177/0271678X15609939

**Published:** 2015-10-23

**Authors:** Joshua D Bernstock, Yang-ja Lee, Luca Peruzzotti-Jametti, Noel Southall, Kory R Johnson, Dragan Maric, Giulio Volpe, Jennifer Kouznetsova, Wei Zheng, Stefano Pluchino, John M Hallenbeck

**Affiliations:** 1 Stroke Branch, National Institute of Neurological Disorders and Stroke, National Institutes of Health (NINDS/NIH), Bethesda, MD, USA; 2 Department of Clinical Neurosciences, Division of Stem Cell Neurobiology, Wellcome Trust-Medical Research Council Stem Cell Institute, University of Cambridge, Cambridge, UK; 3 National Center for Advancing Translational Sciences, National Institutes of Health (NCATS/NIH), Bethesda, MD, USA; 4 Bioinformatics Section, Information Technology & Bioinformatics Program, Division of Intramural Research (DIR), (NINDS/NIH), Bethesda, MD, USA; 5 Flow Cytometry Core Facility, National Institute of Neurological Disorders and Stroke, National Institutes of Health (NINDS/NIH), Bethesda, MD, USA

**Keywords:** High-throughput assay development, miRNA, neuroprotection, SUMO conjugation, translational research

## Abstract

The conjugation/de-conjugation of Small Ubiquitin-like Modifier (SUMO) has been shown to be associated with a diverse set of physiologic/pathologic conditions. The clinical significance and ostensible therapeutic utility offered via the selective control of the global SUMOylation process has become readily apparent in ischemic pathophysiology. Herein, we describe the development of a novel quantitative high-throughput screening (qHTS) system designed to identify small molecules capable of increasing SUMOylation via the regulation/inhibition of members of the microRNA (miRNA)-182 family. This assay employs a SHSY5Y human neuroblastoma cell line stably transfected with a dual firefly-Renilla luciferase reporter system for identification of specific inhibitors of either miR-182 or miR-183. In this study, we have identified small molecules capable of inducing increased global conjugation of SUMO in both SHSY5Y cells and rat E18-derived primary cortical neurons. The protective effects of a number of the identified compounds were confirmed via an *in vitro* ischemic model (oxygen/glucose deprivation). Of note, this assay can be easily repurposed to allow high-throughput analyses of the potential drugability of other relevant miRNA(s) in ischemic pathobiology.

## Introduction

Stroke is one of the most common causes of death and disability worldwide. Due to an aging population, the burden will markedly increase in the coming decades and will be particularly pronounced in developing countries.^1^ Of strokes that occur in the United States, 87% are ischemic, 10% are intracerebral hemorrhagic strokes, whereas 3% are subarachnoid hemorrhages.^1^ Based on this distribution, the majority of research efforts are directed towards the development of interventions/therapeutics capable of targeting the variety of pathophysiological alterations that occur in ischemic stroke.

The continuing failure of clinical trials targeting single mechanisms of neuroprotection in ischemic stroke supports the view of ischemic brain damage as a highly complex multifactorial process, that appears to involve the interplay of many non-dominant effectors. In order to confront the enormous biocomplexity of such network dynamics, it is prudent to focus on plurifunctional targets that affect multiple mechanisms governing homeostasis in states of both natural and acquired tolerance to brain ischemia.

One such candidate that is capable of meeting the aforementioned criteria is that of global SUMOylation. SUMOylation is a form of post-translation modification that operates in states of tolerance and acts to preserve homeostasis under stress via a myriad of beneficial effects in the ischemic network.^2^ Briefly, SUMO, like ubiquitin, is synthesized as an inactive precursor and is processed by SUMO-specific proteases to yield its mature form.^3^ A single heterodimeric E1 enzyme, SAE1/SAE2, serves to initiate conjugation by adenylating SUMO leading to the formation of a covalent thioester E1-SUMO intermediate.^3^ SUMO is then transferred to the catalytic cysteine of the sole E2 conjugase, Ubc9, which alone or in concert with a target specific E3-ligase catalyses the formation of an isopeptide linkage between the C-terminal glycine residue of SUMO and the epsilon amino group of the substrate lysine residue.^3^ SUMO conjugation is balanced via the deconjugative actions of the various SUMO-specific proteases (SENPs).^3,4^ There are three systemically distributed SUMO paralogs in mammals: SUMO-2 and SUMO-3, which are 97% identical and cannot be distinguished by specific antibodies, and SUMO-1 which shares only ∼50% homology with the other paralogs and therefore has distinct immunoreactivity.^3^ SUMOylation has been documented to play a role in numerous processes throughout the cell, including signal transduction, gene expression, chromatin remodeling, and protein translocation.^3,5^

Of particular interest in the context of ischemic pathobiology were the changes (10–30 fold increases) in global SUMOylation levels that have been reported to occur during hibernation torpor in 13-lined ground squirrels (*Ictidomys tridecemlineatus*)^6^ which are one of the most resistant mammals to brain hypoperfusion.^7^ During torpor, these animals significantly reduce their brain blood flow levels to roughly 10% of their baseline, yet upon arousal show no evidence of cellular damage and/or functional deficits despite prolonged exposure to perfusion levels characteristic of the “ischemic core”.^7,8^ Further, *in vitro* work conducted in both immortalized cell lines and primary cortical neuronal cultures exposed to periods of oxygen and glucose deprivation (OGD) confirmed that increases in global SUMOylation are in fact cytoprotective.^9^ We went on to show that transgenic mice that overexpress Ubc9 do in fact increase global SUMOylation levels and confer a corresponding level of resistance to brain ischemia.^10,11^ In so doing, we established that the level of global SUMOylation is directly proportional to the level of cytoprotection in preclinical models of stroke.^10^

Additional work has since focused on elucidating the molecular mechanisms that control the levels of global SUMOylation. The goal of this effort is to develop methods capable of boosting global SUMOylation to those levels seen in hibernating animals and to test whether comparable cytoprotection can be reproduced in stroke models. To this end, we have recently identified a series of microRNAs serving as regulators of both global SUMOylation and global post-translational modification by other ubiquitin-like modifiers (ULMs) including NEDD8, ISG15, UFM1 and FUB1 (all of which were significantly increased in the brains of hibernating ground squirrels during torpor).^12^ This report was the first to link the natural tolerance to brain ischemia, witnessed in hibernators, to multimodal regulation by miRNAs. Analyses established that the miR-200 family (miR-200 a,b,c/miR-141/miR-429) and the miR-182 family (miR-182/miR-183/miR-96) were consistently depressed in the brain during the torpor phase as compared to active animals.^12^ We showed that the inhibition of the miR-200 family and/or miR-182 family in SHSY5Y cells increased global protein conjugation by the abovementioned ULMs, and in so doing made these cells more resistant to OGD-induced cell death.^12^ Collectively, such evidence suggests that augmentation of global SUMOylation may potentially be harnessed and exploited for the protection of vulnerable ischemic tissue through the manipulation of miRNA.

Herein we describe the development of a novel qHTS assay designed to uncover small molecules that increase global SUMOylation via inhibition of the miR-182 family. The validity of the assay was confirmed by immunoblotting. Of note, a select number of compounds were capable of inducing protection during OGD in both SYSH5Y cells and E18 primary cortical neurons thereby confirming the functional utility of this assay.

## Materials and methods

### Generation of dual-luciferase miRNA target expression constructs

**Figure 1.**
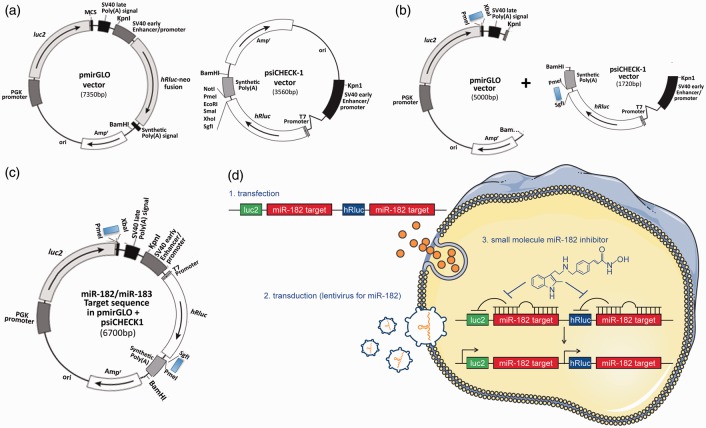
Generation of dual-luciferase miRNA target expression constructs and their stable transfectants. (a) Commercially available original vectors, pmirGLO and psiCHECK-1, which were designed to quantitatively evaluate miRNA activity via the insertion of miRNA target sites on the 3′ UTR of the firefly gene (*luc2*) (pmirGLO) or 3′ UTR of the Renilla gene (*hRluc*) (psiCHECK-1). (b) Insertion of annealed oligonucleotide pairs, which contain the miR-182 (or miR-183) target sequence with appropriate restriction sites (PmeI and XbaI for pmirGLO, SgfI and PmeI for psiCHECK-1) downstream of *luc2* in the pmirGLO and downstream of *hRluc* in the psiCHECK-1. These vectors were digested with KpnI and BamHI and the fragments which contained the reporter units were isolated/ligated. (c) The final construct consists of a dual reporter system utilizing both firefly and Renilla luciferase. (d) (1) Using SHSY5Y cells, stable transfectants of the engineered reporter constructs were created (specific for either miR-182 [represented] or miR-183). (2) In order to maintain minimal basal levels of activity of both luciferases, the stable host cells were transduced with lentiviral particles containing miR-182 (or miR-183) shMIMIC microRNAs. (3) These stable transfectants (miR-182 or miR-183 target sequence in pmirGLO/psiCHECK1 plus lentiviral particles containing miR-182 or miR-183 shMIMIC) were usable for high-throughput screens. The final stable reporter cell line was designed to identify small compounds (e.g. HDAC inhibitor Panobinostat) that inhibit miR-182 (and/or miR-183) generation or function, thereby resulting in the activation of the luciferases (both firefly and Renilla).

The pmirGLO (Promega (Madison, WI, USA)) and the psiCHECK-1 (Promega) vectors were designed to quantitatively evaluate miRNA activity via the insertion of specific target sites into the 3′ untranslated region (UTR) of the firefly (pmirGLO vector) or Renilla (psiCHECK-1) luciferase gene mRNA. Starting from these two vectors, we built a dual reporter construct with the miR-182 (or miR-183) target sequence (Figure 1 and Supplementary Figure 1), so that the presence of mature miR-182 or miR-183 would lead to a decrease in luciferase (both firefly and Renilla) signal, enabling the detection of putative miR-182 (or miR-183) levels. Post-construction, we examined whether these constructs would work as had been predicted. We transfected SHSY5Y cells transiently with these constructs along with either negative control miRNA or miR-182 (or miR-183) mimics (miRIDIAN micro RNA Negative control or Mimics, Thermo Fisher Scientific (Waltham, MA, USA)) and measured luciferase activities. As shown in Supplementary Figure 2a, increased miR-182 (or -183) levels induced via the transfection of mimics significantly depressed both firefly and Renilla luciferase activity. Next we contrived SHSY5Y stable transfectants of the engineered constructs. The established stable transfectants responded well to both miR-182 or miR-183 mimics (i.e. the transfection of these mimics caused the depression of both firefly and Renilla luciferase acitivities in each cell line (Supplementary Figure 2b)). Of note, the endogenous levels of both miR-182 and miR-183 are quite low in SHSY5Y cells and thus the basal levels of luciferase activities are quite high (Supplementary Figure 2a and b). In order to maintain minimal basal levels of luciferase activities, we transduced these stable cell lines with lentiviral particles containing miR-182 (or miR-183) shMIMIC microRNAs (Thermo Fisher Scientific), and in so doing established cell lines that constitutively expressed these miRNAs via the selective pressure of puromycin. We then examined whether these stable transfectants (miR-182 or miR-183 target sequence in pmirGLO/psiCHECK1 plus lentiviral particles containing miR-182 or miR-183 shMIMIC) were usable for high-throughput screens. The final stable reporter cell line was designed to identify small compounds that inhibit miR-182 (and/or miR-183) generation or function, thereby resulting in the activation of the luciferases (both firefly and Renilla). We used a miR-182 (or miR-183) inhibitor (miRIDIAN microRNA hairpin inhibitor, Thermo Fisher Scientific) as a positive control and non-specific miRNA (miRIDIAN microRNA Negative Control, Thermo Fisher Scientific) as a negative control. As shown in Supplementary Figure 2(c), the basal level of luciferase activities (both firefly and Renilla) are very low in comparison to negative controls in Supplementary Figure 2(a) and (b) and the activation by the miR-182 (or miR-183) inhibitors were substantial. Calculated Z-factors, which represent a well-established quantitative measure of the quality of an assay, were 0.61 for miR-182/firefly, 0.5 for miR-182/Renilla, 0.47 for miR-183/firefly, and 0.66 for miR-183/Renilla. Being that a Z-factor between 0.5 and 1.0 corresponds to an excellent assay, our assay system qualifies as having sufficient sensitivity for high-throughput screening.

### Cell culture and transfection/transduction

The human neuroblastoma cell line SHSY5Y (ATCC (Manassas, VA, USA)) and its stable derivatives (stable reporter cell lines) were cultured in DMEM supplemented with 10% FBS, 100 U/mL penicillin and 100 µg/mL streptomycin (pen/strep) at 37℃ with 5% CO_2_. Cortical neurons were isolated from E18 embryos of Sprague-Dawley rats in accordance with the policies set forth by the ACUC (Animal Care and Use Committee) of NINDS and experiments were performed according to ACUC, NIH, and ARRIVE guidelines (http://www.nc3rs.org/ARRIVE). Cells were plated on poly-l-lysine coated plates and cultured in Neurobasal media (Gibco (Waltham, MA, USA)) supplemented with B27 (Gibco), pen/strep as previously described^9^; cells were used after seven days in culture. Transfection of SHSY5Y cells was performed using electroporation with nucleofector (Amaxa (Basel, Switzerland)) for plasmid constructs and Lipofectamine 2000 (Invitrogen (Waltham, MA, USA)) for miR mimics or inhibitors per the manufacturers’ instructions. Transductions of shMIMIC Lentiviral miRNA particles were performed at low multiplicities of infection (MOI) (i.e. 0.3) according to the manufacturer’s (Thermo Fisher Scientific) instructions in the media devoid of serum and antibiotics.

### Assay for firefly and Renilla luciferase activities

We used ‘Dual-Glo Luciferase Assay System (Promega)’ to measure luciferase (firefly and Renilla) activities according to the manufacturer’s instructions. Briefly, after cells were transfected with the miRNA mimics/inhibitors or treated with small molecules, Dual-Glo Luciferase Assay reagent was added to each well at a volume equal to that of the culture (i.e. 80 µl in 96-well plate; 4 µl in 1536-well plate), incubated for 10 min at room temperature and the luminescence measured (firefly). Then, the Dual-Glo Stop and Glo reagent was added to the plate (same volume as the first reagent), incubated for another 10 min at room temperature, and the luminescence measured (Renilla). Luminescence in 96-well plates was measured using a LB 960 Centro (Berthold Technology (Oak Ridge, TN, USA)), while luminescence in 1536 plates was measured via the ViewLux plate reader (PerkinElmer (Waltham, MA, USA)).

### qHTS assay and viability

For each cell line tested, a total of 2000 cells per well in 4 µL of media were dispensed using a Multidrop Combi dispenser (Thermo Fisher Scientific) and a small cassette into barcoded 1536-well flat-bottom white (Corning (Corning, NY, USA)) collagen-coated plates. After a 24 h incubation, library compounds and controls were added to assay plates at a volume of 23 nL/well via a NX-TR pintool station (Wako Scientific Solutions (San Diego, CA, USA)), and the plates were incubated further (another 24 h). Firefly and Renilla luminescence outputs were measured sequentially using the Dual-Glo Luciferase Assay System (Promega) and the ViewLux plate reader (PerkinElmer). The assay’s performance was stable throughout the screen. The activity of each compound was normalized to control wells (DMSO alone), which were included on each plate. Cells treated with DMSO alone were defined as having 0% activity. Of note, the following libraries were screened: the library of pharmacologically active compounds (LOPAC),^13^ MIPE^14^ and the NIH Chemical Genomics Center (NCGC) Pharmaceutical Collection (NPC).^15^

### Western blot analysis

Whole cell lysates were prepared and subjected to SDS-PAGE as described previously.^9^ The antibodies used in this study were: anti-SUMO-1 (rabbit polyclonal, in house), ant-SUMO-2,3 (rabbit polyclonal, in house), anti-Ubc9 (rabbit monoclonal, Abcam (Cambridge, UK)) and anti-β-actin (mouse monoclonal, Sigma (St. Louis, MO, USA)). Intensities of bands were analysed using Image-J (NIH (Bethesda, MD, USA)). In order to measure SUMO conjugation levels, the region corresponding to molecular weights above 100 kDa in each lane was cropped and the total intensity was analysed. The densities were normalized with corresponding actin levels and expressed as the ratio to control (DMSO alone).

### OGD and the assessment of cell death

OGD for SHSY5Y and rat cortical neurons were performed as described previously.^6,9^ We subjected cells with or without drugs to OGD for 15 h (SHSY5Y cells) or 5 h (cortical neurons) followed by the restoration of oxygen/glucose (ROG) for 6 h (SHSY5Y) or 16 h (cortical neurons). The duration of OGD and ROG was determined by our previous studies.^6,9^ Cell death was assessed via nuclear staining with Hoechst 33342 and propidium iodide (PI) followed by fluorescence-activated cell sorting (FACS) analysis.^6,9^ Typically, 1 × 10^5^ cells were analysed. The percentage of total cell death (both apoptotic and necrotic) was calculated by taking the difference of viable cell populations between non-OGD and OGD-subjected, and dividing it by the viable cell population of non-OGD, with the understanding that some of compounds themselves may have an effect on cell viability without OGD (i.e. toxicity at time points > 13 h). The compounds were all dissolved in DMSO and were subsequently diluted to attain a final DMSO concentration of 0.1% in all experiments, and 0.1% DMSO alone, without drug was used as our OGD/ROG control. We normalized the calculated cell deaths to the 0.1% DMSO control within each experiment. Cell death was also assessed by measuring LDH release according to the manufacturer’s directions (Abcam).

### Quantification of neuronal apoptosis/death via microscopic histology

Primary cortical neurons were plated at a density of 3 × 10^5^ on poly-l-lysine-coated Lab-Tek chamber slides (Nalge Nunc International (Waltham, MA, USA)) for the assessment of neuronal apoptosis/death. After OGD or OGD/ROG, neurons were fixed/subsequently stained with terminal deoxy-nucleotide transferase dUTP nick end labeling (TUNEL) and a rabbit anti-β-tubulin (Covance (Princeton, NJ, USA) MRB-435 P) antibody, followed by an anti-rabbit Alexa Fluor 647-conjugated secondary antibody. Nuclei were counterstained with 4′,6-diamidino-2-phenylindole (DAPI). Briefly, for TUNEL staining (APO-BrdU™ TUNEL Assay, Thermo Fisher Scientific), 70% ethanol-fixed cells were incubated in the provided DNA-labelling solution which contained TdT and BrdUTP for 1 h at 37℃. After incubating the cells with the staining solution (Alexa Fluor 488 dye-labeled anti-BrdU antibody) for 30 min at RT, the PI/RNase A staining buffer was applied for an additional 30 min. TUNEL^+^ cells were then manually counted using microphotographs collected at six random regions of interest (ROIs). Data are expressed as mean percentage ± standard error (SE) of TUNEL^+^ cells over DAPI.

### Analysis of dendritic arborizations and spines

Primary cortical neurons were plated at a density of 1.5 × 10^5^ cells in chamber slides for the assessment of dendritic arborizations and spine density. After OGD or OGD/ROG, neurons were fixed/stained using a chicken anti-microtubule-associated protein 2 (MAP2) (Abcam ab5392) and a mouse anti-postsynaptic density protein 95 (PSD95) (Abcam ab2723) primary antibodies, followed by an anti-chicken Alexa Fluor 488-conjugated and an anti-mouse Alexa Fluor 568-conjugated secondary antibody respectively. Nuclei were counterstained with DAPI. For the initial assessment of MAP2 immunoreactivity, immunofluorescence stainings were evaluated using a CCD camera/fluorescence microscope; *n* = 6 equally distributed ROIs were acquired via a 20x objective lens for analysis. Data are expressed as MAP2^+^ area (mm^2^) ± SE over DAPI. MAP2^+^/PSD95^+^ neurons were then analysed in more detail using a 63x objective lens for the evaluation of dendritic arborizations and spines. Criteria for inclusion within the analyses were the following: (i) the neuron had to be well stained; (ii) the neuron had to be in full view, (i.e. neither obscured/obstructed by overlapping dendrites from other neurons); and (iii) the neuron had to contain intact dendritic arborizations (i.e. not display any obvious signs of degeneration).^16^ The first four neurons from each experimental condition that fit the abovementioned criteria were reconstructed by following the dendrites through the *z*-axis, and the length of each dendritic branch was determined using Sholl and Branch analysis via StereoInvestigator software (MicroBrightField (Williston, VT, USA)), as has been previously described.^17^ For statistical analysis, we used a standard software package (GraphPad Prism version 4.0). Histological data were evaluated by unpaired two-tailed *t*-tests (for comparisons between two groups) and by one-way ANOVA followed by Newman–Keuls tests for post hoc analysis (for comparison amongst ≥ three groups). To test for differences in dendritic length (Sholl analysis), a two-way ANOVA, followed by Tukey’s post hoc test, was performed. Values of *p* ≤ 0.05 were deemed to be significant.

### Active compound-gene (protein) interaction and stroke enrichment enquiry

The Ingenuity Pathway Analysis (IPA) tool was used (www.ingenuity.com). Compounds by name were entered into IPA and all known gene (protein) interactions supported by IPA returned. These interactions were summarized in IPA via a network view and enumerated/compared across compounds via a bar plot using Microsoft Excel. IPA was also used to identify biological pathways and functions enriched with proteins having interactions with each compound, by compound. Enrichment results were summarized both by heat map using R (www.cran.r-project.org) and by bar plot using Microsoft Excel. Lastly, IPA was queried for all proteins having association with stroke. These stroke-associated proteins were then intersected with proteins having interaction with each compound by compound and an enrichment *p*-value calculated for the intersection via Chi-square test with Yates correction using GraphPad Prism.

### qHTS data analysis

Plate-based qHTS data were normalized and concentration–effect relationships derived using in-house developed software.^18^ The activity of each compound was normalized to vehicle control wells (DMSO), which were included on each plate, and reported as an absolute percentage change in signal relative to these control wells. EC50 values were obtained by data fit using a residual error minimization algorithm with automatic outlier determination to a four-parameter Hill equation as the dose–response model. Concentration–effect relationships (CERs) were categorized by fit quality (r2), response magnitude, and degree of measured activity.^18^

## Results

### Quantitative high-throughput screening (qHTS) via a dual luciferase reporter assay for the identification of inhibitors of miRNA 182/183

**Figure 2.**
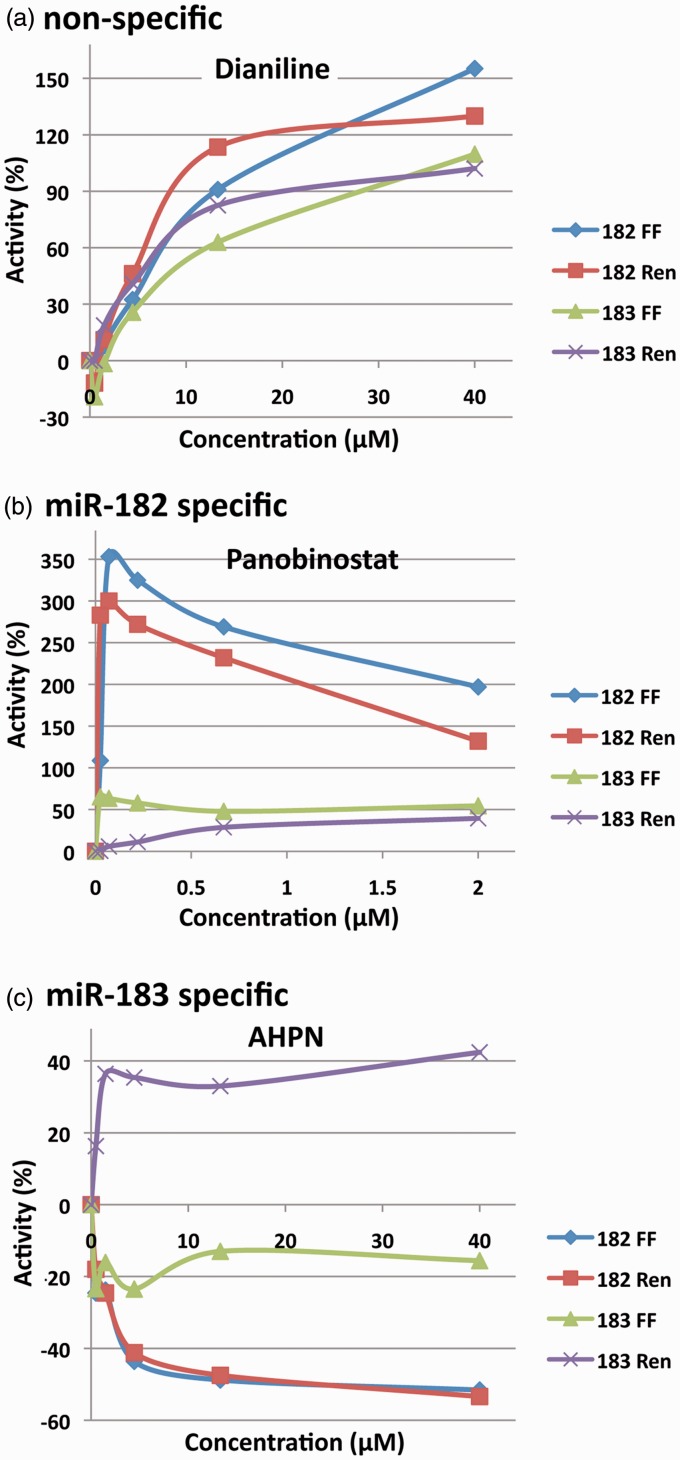
Dose–response curves. (a) The non-specific response of Dianiline across five concentrations. (b) The miRNA-182 specific response of Panobinostat. (c) The miRNA-183 specific response of AHPN.

The ultimate goal of this work was to develop a system capable of identifying molecular entities (MEs)/active pharmaceutical ingredients (APIs) capable of upregulating global SUMOylation through the inhibition of miRNAs 182 and/or 183. To minimize interference and increase our confidence in hits ascertained during screening, we designed constructs that contain two different reporters (dual reporter system), firefly luciferase and Renilla luciferase (Figure 1), which are not homologous and therefore have unrelated bioluminescent properties. We confirmed that the presence of mature miR-182 or miR-183 would lead to a decrease in luciferase (both firefly and Renilla) signal, enabling the detection of putative miR-182 (or miR-183) levels (Supplementary Figure 2). We then established cell lines which stably expressed these constructs as described in Figure 1, and used them for the screening of small molecule libraries in a 1536 well format. From a total of 4489 compounds screened, 120 compounds were initially identified in the course of the primary screening process. These 120 compounds were subsequently selected and re-screened for validation through the use of confirmatory assays. From the follow-up screening, 21 active compounds (listed in Table 1) were confirmed based on their activities in both firefly and Renilla luminescence assays and were taken forward for further study/characterization. We note that most confirmed compounds do not give equivalent percentage changes in signal in both channels, perhaps due to a difference in sensitivity in the detection methodology for these readouts (Figure 2 and Supplementary Figure 3).

**Table 1.** Molecular entities – libraries of origin and regulatory status.

	Molecular entity	Library	Status
1	Romidepsin	NPC	Approved drug
2	Panobinostat	NPC	Clinical testing
3	Entinostat	NPC	Clinical testing
4	Belinostat	NPC	Approved drug
5	Pracinostat	NPC	Approved drug
6	Fosmidomycin	NPC	Clinical testing
7	NCGC00185916	NPC	Preclinical testing
8	Licofelone	NPC	Clinical testing
9	Motesanib	NPC	Clinical testing
10	Orotic acid	NPC	Clinical testing
11	JWH-015	LOPAC	Preclinical testing
12	5-Azacitidine	LOPAC	Approved drug
13	AHPN	NPC	Preclinical testing
14	Lenalidomide	NPC	Approved drug
15	Vatalanib	NPC	Clinical testing
16	VX-702;KVK-702	NPC	Clinical testing
17	Dianiline	NPC	Preclinical testing
18	Diazoxide	NPC	Approved drug
19	Telmisartan	NPC	Approved drug
20	JQ1	NPC	Preclinical testing
21	TW-37	NPC	Preclinical testing

### Active compounds identified during the primary screen induced SUMO conjugation in SHSY5Y cells and rat E18 primary cortical neurons

**Figure 3.**
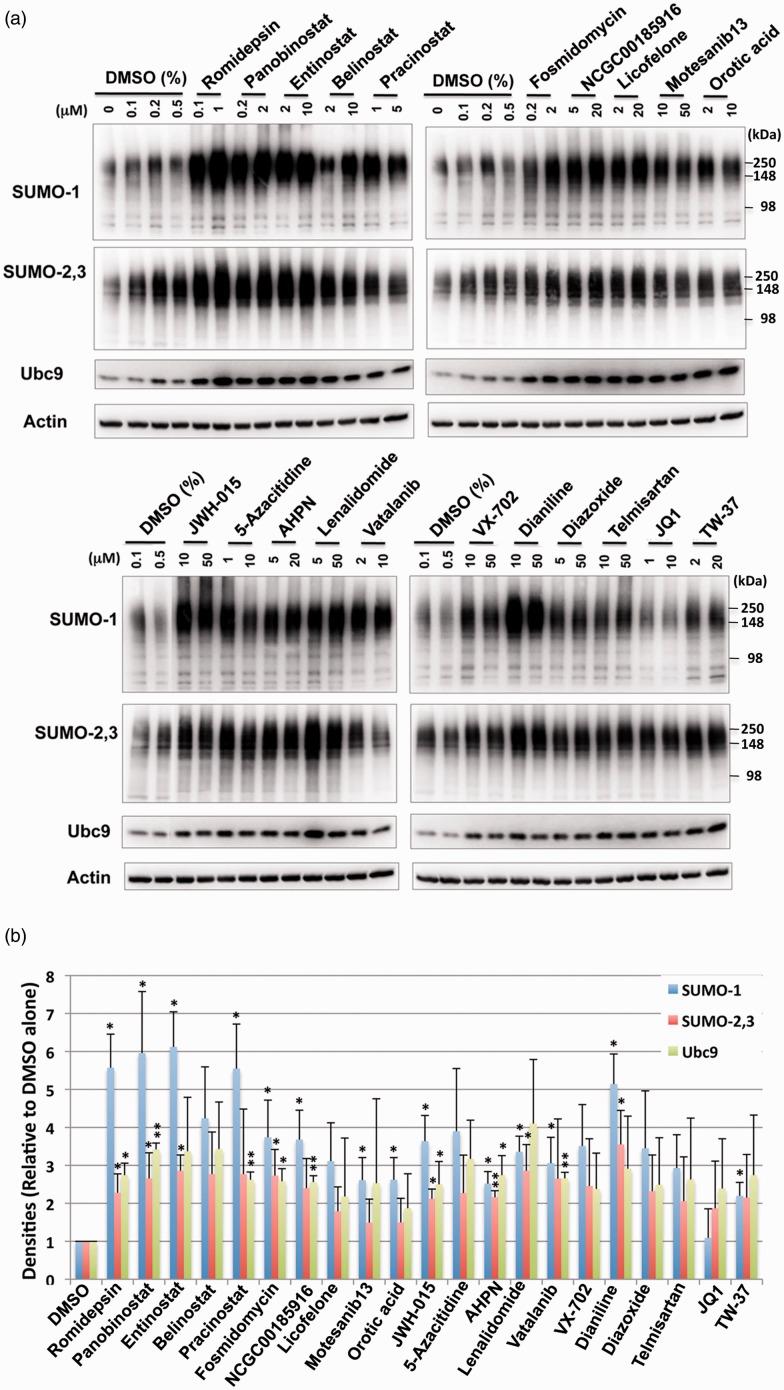
Treatments with small molecules identified by qHTS increase the levels of SUMO conjugation and the Ubc9 conjugase in SHSY5Y parent cells. (a) Representative immunoblots of high molecular weight (>100 kDa) SUMO-1 and SUMO-2,3 conjugates and the Ubc9 protein in the total cell lysates from SHSY5Y cells treated with various compounds with indicated concentrations for 13.5 h. (b) Quantitative analyses of the conjugates and Ubc9 from three independent experiments. High molecular weight SUMO-1 or SUMO-2,3 conjugates (>100 kDa) were cropped in each lane and the total intensity measured. The densities were normalized to corresponding actin levels and expressed as the ratio to control (DMSO alone). Data represent the mean+/−standard deviation of three independent experiments. **p < 0.01, *p < 0.05 compared to DMSO control.

**Figure 4.**
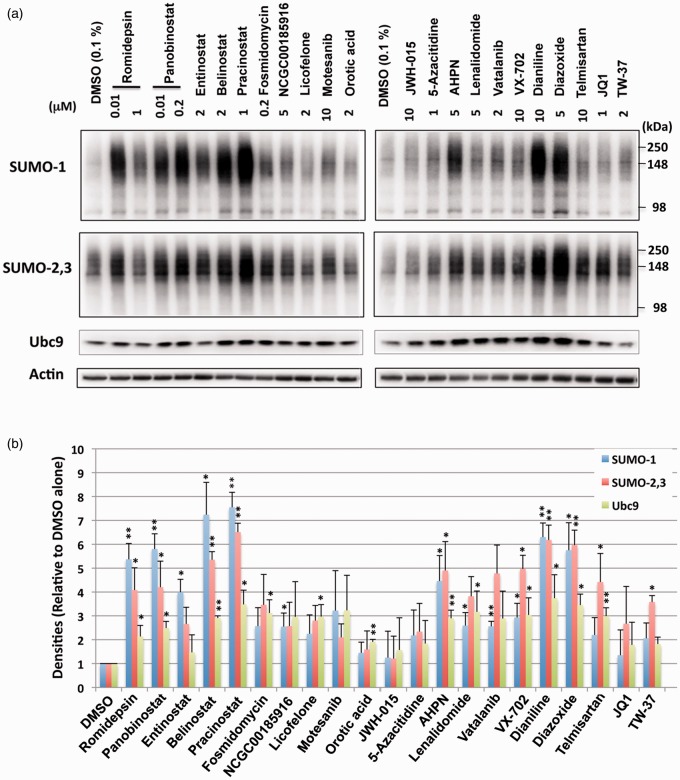
The effect of small molecules identified by qHTS on SUMO conjugation and Ubc9 levels in E18 rat cortical neurons. (a) Representative immunoblots of high molecular weight (>100 kDa) SUMO-1 and SUMO-2,3 conjugates and the Ubc9 conjugase in the total cell lysates from rat E18 primary cortical neurons treated with various compounds with the indicated concentrations for 13.5 h. (b) Quantitative analyses of the conjugates and Ubc9 from three independent experiments. Data represent the mean+/−standard deviation of three independent experiments. **p < 0.01, *p < 0.05 compared to DMSO control.

To determine the effect of the active compounds identified via the primary screen on the induction of SUMO conjugation, the 21 compounds were tested in orthogonal cell-based assays with SHSY5Y cells. The compounds were incubated with SHSY5Y cells for 13.5 h at both a low and high concentration extrapolated from the concentration–response curves of the confirmatory assay. As shown in Figure 3, immunoblotting effectively demonstrated that the majority of the compounds identified were indeed capable of upregulating SUMO1 and SUMO-2/3 conjugation, thereby confirming the biologic validity of the positive hits resulting from the qHTS assay. Further, it was noted that many of the compounds seemed to increase the levels of the sole SUMO E2 conjugase Ubc9 as well (Figure 3). To examine whether the upregulation of global SUMO conjugation by these compounds in SHSY5Y cells (stable derivatives of which we used for the screening) were cell type specific, or not, we next examined the effect of the compounds on the SUMOylation levels of primary cortical neurons isolated from rat embryos. Since the lower dose of the majority of the compounds had a similar effect when compared to the higher dose in SHSY5Y cells (Figure 3), we explored the lower/physiologically compatible dose in primary cortical neurons. As shown in Figure 4, most compounds were capable of increasing the levels of global SUMO conjugation in primary cortical neurons. Interestingly, the levels of global SUMOylation induced did vary from compound to compound between the SHSY5Y cell line and the primary cortical neurons (Figures 3 and 4). To exclude the confounding contributions of compound cytotoxicity, a cell viability assay was performed in parallel with the SUMO conjugation assay. We found that the increases in global SUMOylation were unrelated to a cellular stress response or cell death as compound cytotoxicity was not observed; critically, cells were treated with the compounds for an equivalent amount of time (Supplementary Figure 4).

### qHTS identified active compounds are capable of inducing protection against oxygen/glucose deprivation (OGD)/restoration of oxygen/glucose (ROG)

**Figure 5.**
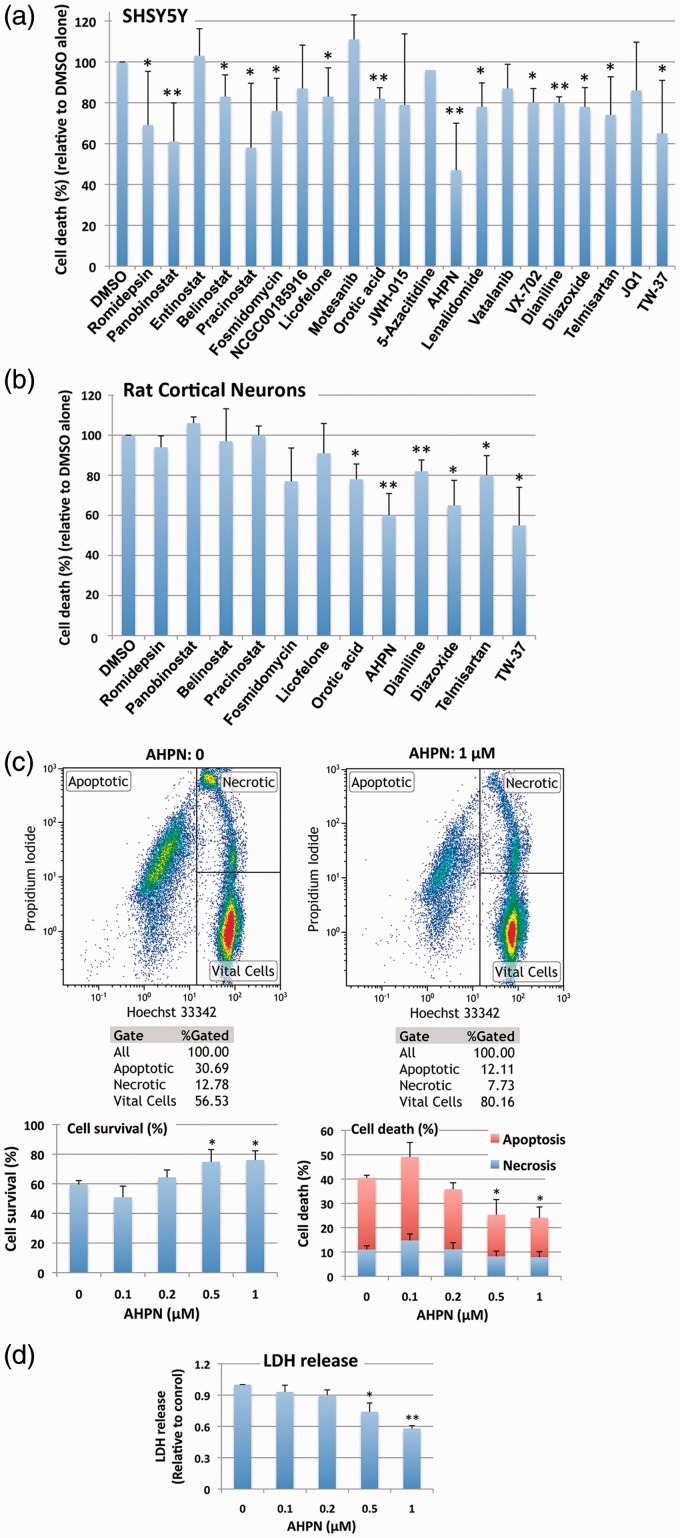
The effect of small molecules identified by qHTS on OGD/ROG-induced cell death in SHSY5Y cells and E18 rat cortical neurons. After OGD/ROG exposure (OGD/ROG: 16 h/5 h for SHSY5Y, 5 h/16 h for cortical neurons) in the presence or absence of the small molecules indicated, cell death/survival was assessed via nuclear staining with Hoechst 33342 and propidium iodide (PI) followed by FACS analyses. The percentage of total cell death (both apoptotic and necrotic) was calculated and recorded vs the percentage of 0.1% DMSO control cell death in each experiment. (a) SHSY5Y cells. (b) Rat cortical neurons. (c) Increasing concentrations of AHPN displayed statistically significant increases in viability and decreases in cell death after OGD/ROG as measured by FACS analysis. Upper panels: representative dot plots without AHPN (left) and with 1 µM AHPN (right) with numbers in each population (left, apoptotic cells; upper right, necrotic cells; lower right, viable cells); lower panels: Quantitative analyses of cell survival (left) and cell death (right). (d) Increasing concentrations of AHPN displayed statistically significant decreases in cell death as measured by LDH release. Data represent the means ± standard deviation of three independent experiments. **p < 0.01, *p < 0.05 compared with OGD/ROG without AHPN by student's t-test.

**Figure 6.**
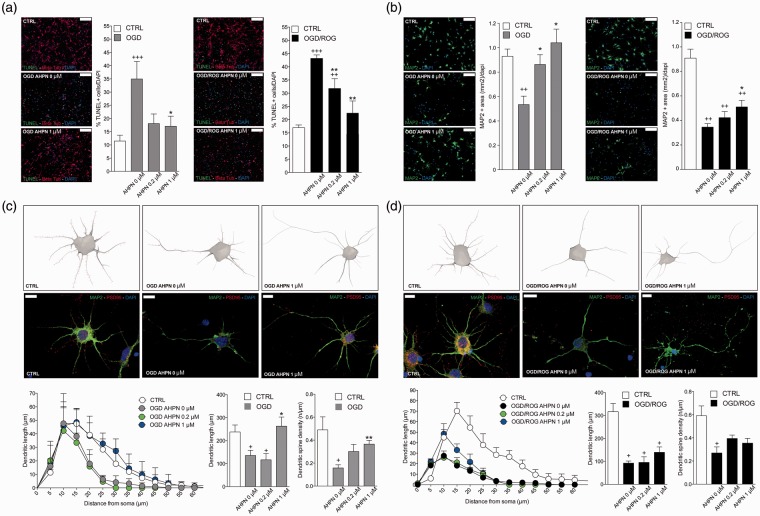
AHPN reduces ischemic neuronal apoptosis and preserves dendritic/synaptic integrity *in vitro*. (a) Representative images of TUNEL^+^ (in green) and β-tubulin^+^ (in red) neurons and quantitative analysis. Nuclei are counterstained with DAPI (blue). Scale bars: 100 µm. Data are means ± standard error. *p ≤ 0.05, compared with OGD and OGD/ROG controls, respectively. ^+++^*p* ≤ 0.001, ^++^*p* ≤ 0.01, compared with non-hypoxic CTRL neurons; **p ≤ 0.01, *p ≤ 0.05, compared with OGD or OGD/ROG controls. (b) Representative microphotographs of primary cortical neurons dendrites stained for MAP2 (green) and corresponding quantification. Nuclei are counterstained with DAPI (blue). Scale bars: 100 µm. Note the dose-dependent increase of MAP2 expression induced by AHPN in both OGD and OGD/ROG treated neurons. ^++^*p* ≤ 0.01 compared with non-hypoxic CTRL neurons; *p ≤ 0.05, compared with OGD or OGD/ROG controls. (c,d) Representative reconstructions and microphotographs of OGD (c) and OGD/ROG (d) primary cortical neurons treated with AHPN. Dendrites are stained with MAP2 (green), while spines are stained with PSD95 (red). Nuclei are counterstained with DAPI (blue). Scale bars: 10 µm. Sholl analysis of dendritic length with nested concentric spheres centred at the cell soma and with a gradually increasing radius (5 µm) showing cumulative dendritic length in the function of cell soma distance, the integrated total dendritic length, and the spine density of primary cortical neurons. ^+^*p* ≤ 0.05, compared with non-hypoxic CTRL neurons, **p ≤ 0.01, *p ≤ 0.05, compared with OGD or OGD/ROG controls.

The ultimate goal of this qHTS was to characterize a novel method capable of uncovering small molecules that might be used for the treatment of ischemic stroke. The 21 compounds were consequently tested for their putative efficacy in protecting SHSY5Y cells from OGD followed by ROG *in vitro*. Concentrations were chosen based on the aforementioned qHTS (Supplementary Table 1). As shown in Figure 5(a), most compounds induced protection from OGD/ROG-induced cell death in SHSY5Y cells, with Panobinostat (a histone deacetylase [HDAC] inhibitor) and 6-[3-adamantyl-4-hydroxyphenyl]-2-napthalene carboxylic acid (AHPN), a synthetic retinoid, being the most effective. We then tested the active compounds that displayed statistically significant effects in E18 primary cortical neurons. We found that far fewer compounds displayed protective effects against OGD/ROG in E18 primary cortical neurons as compared to SHSY5Y cells, but again AHPN was noted to be effective (Figure 5b). Being that AHPN was found to be the most effective compound in protecting both SHSY5Y and E18 primary cortical neurons from OGD/ROG-induced cell death, we decided to further characterize its effects on primary cortical neurons. As previously reported, OGD/ROG treatment causes both apoptosis/necrosis and these populations can be distinguished via FACS.^9^ As shown in Figure 5(c), 5 h OGD followed by 16 h ROG caused ∼40% cell death (30% apoptosis/10% necrosis) in untreated cells (i.e. DMSO alone) whilst increasing concentrations of AHPN during OGD/ROG provided statistically significant decreases in amounts of cell death. While both apoptosis and necrosis levels were decreased, AHPN was most effective in decreasing the number of apoptotic cells. Total cell death after OGD/ROG was also assessed by LDH release (Figure 5d). In addition, we analysed how AHPN protects primary cortical neurons after 5 h OGD, or 5 h OGD followed by 16 h ROG via both TUNEL and MAP2/PSD95 staining. We found that 1 µM AHPN induced a significant reduction in TUNEL^+^ neurons (a marker of cell apoptosis/death) when compared with both control neurons exposed to OGD and OGD/ROG (Figure 6a). Interestingly, the lowest dose of AHPN (0.2 µM) also displayed a significant effect on the survival of OGD/ROG neurons (Figure 6a). Critically, both *in vivo* ischemia and *in vitro* OGD have been shown to induce alterations of dendrites (i.e. early degeneration^19^ and a compensatory outgrowth^20^). As such, we decided to examine MAP2 immunoreactivity in the surviving cortical neurons after OGD and OGD/ROG, being that MAP2 is a cytoskeletal phosphoprotein that provides scaffolding within dendrites, particularly near spines.^19^ Interestingly, we found that AHPN was capable of preventing a decrease in MAP2 intensity after OGD and OGD/ROG in a dose-dependent manner (Figure 6b). We then examined the dendritic arborization of primary cortical neurons and focused our attention on PSD 95 positive spines (Figure 6c and d). Sholl analysis performed on the neuronal three-dimensional (3D) reconstructions confirmed that the total dendritic length was different among groups with a significant decrease in dendritic arborizations occurring after exposure to OGD and OGD/ROG as compared to non-hypoxic controls. Interestingly, AHPN at 1 µM was capable of significantly inhibiting the decrease in neurons subjected to OGD (Figure 6c) while it had no clear effect on neurons exposed to both OGD/ROG (Figure 6d).

### Gene/protein expression and biological pathways perturbed by active compounds

**Figure 7.**
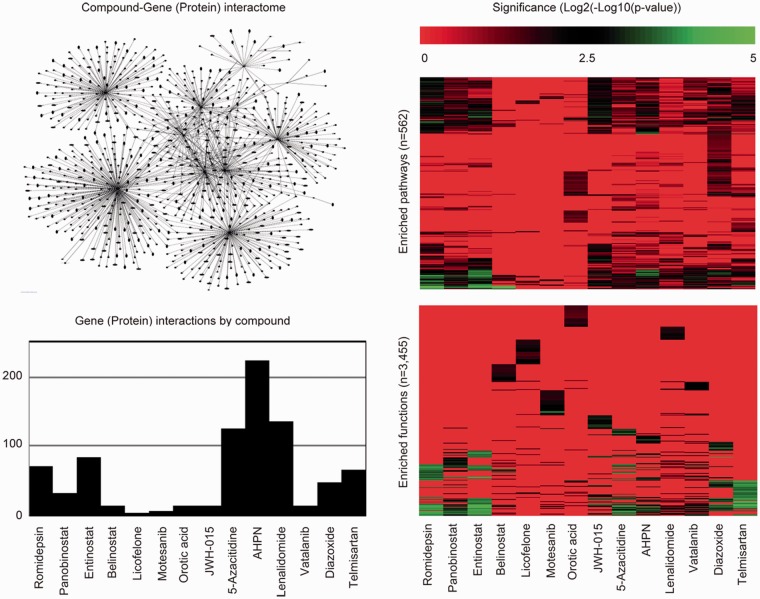
Off-target enquiry. The Ingenuity Pathway Analysis tool (IPA) was used to query all gene (protein) interactions by compound (www.ingenuity.com). These interactions were summarized in two ways. First, a network was constructed across compounds to demonstrate that no exclusive gene (protein) interactions exist (upper left plot). Second, a bar plot was constructed to describe that the number of gene (protein) interactions by compound is unbalanced (lower left plot). To gauge and compare compound impact on biological pathways and functions, IPA was used to report enrichment *p*-values (Fisher’s exact test) for 562 pathways (upper right plot) and 3455 functions (lower right plot).

To explore the potential mechanisms of the active compounds, the Ingenuity Pathway Analysis (IPA) knowledgebase was used to query all gene (protein) interactions contained therein having association with the compounds listed in Figure 7. Summarization and exploration of these interactions via a network diagram revealed that certain compounds have a greater number of interactions than others; suggesting the perturbation potential at the molecular level for these compounds may too be greater. To enumerate and compare the difference in number of interactions across compounds, a bar plot was constructed. By this plot, AHPN has the greatest number of interactions compared to licofelone, a dual COX/LOX inhibitor, which has the fewest. To further elucidate a compound’s perturbation potential at the molecular level, IPA was used again to identify those biological pathways and functions supported in IPA that are significantly enriched (Fisher's exact test *p* < 0.05) for the genes (proteins) associated with each compound. To enumerate and compare the difference in number of significantly enriched biological pathways and functions across compounds, bar plots were constructed. From these plots, compounds having the greatest number of significantly enriched biological pathways (e.g. AHPN, romidepsin, and entinostat) and functions (e.g. romidepsin, entinostat, and telmisartan) could be readily identified and argued to have the greatest perturbation potential at the biological pathway and function levels. Of particular interest is Supplementary Table 2, which describes the number of associated genes (proteins) per compound in IPA, the number of genes (proteins) associated with stroke in IPA, and whether the intersection via Chi-square test is significant (Yates corrected *p* < 0.05) or not. Per the results, 64% of the active compounds (9/14) were found to be significantly enriched for genes (proteins) associated with stroke.

## Discussion

Therapeutic options for ischemic brain injury are limited. As such, the research presented herein seeks to highlight the development of a novel cell-based qHTS system designed to streamline the clinical development and translation of previously approved drugs that may ultimately be repurposed for the treatment of ischemic stroke. In an effort to elucidate targets capable of providing the plurifunctional cytoprotection needed to overcome the inherent complexities of stroke pathobiology, mechanisms of tolerance were examined in the hibernating 13-lined ground squirrel. *I. tridecemlineatus* has an extraordinary capacity to withstand prolonged and profound reductions of blood flow and oxygen delivery to brain without incurring any cellular damage.^7^ One of the underlying molecular means allowing this multifactorial cytoprotection to unfold is that of global SUMOylation.^6,9^ The identification of miRNAs 182 and 183 (reported functions reviewed by Lee et al.^12^) as an endogenous mechanism, which in part controls global SUMOylation, pushed us to identify small molecules capable of regulating this form of post-translational modification via miRNA as novel targets for development of stroke therapies.^12^

MicroRNAs are key players within gene regulatory networks and modulate multimodal gene expression by binding to complementary sequences in target mRNAs, and as such are critically positioned to influence network dynamics/outcomes. One miRNA usually targets more than one hundred genes^21^ with interactome hubs and downstream signaling components (e.g. transcription factors) typically regulated by more miRNAs than other nodes within a given network.^22,23^ Of note, miRNA binding may suspend and/or permanently repress the translation of a given mRNA transcript,^24,25^ thereby altering post-transcriptional gene profiles.

Drugs that directly modulate a single molecular target have come to be understood as insufficient for the cytoprotective treatment of ischemic stroke, unless such a target is itself critically positioned to influence a network.^22^ If one seeks to combat the pathobiology underlying complex and/or polygenic disease processes, the design of a new generation of efficacious drugs must be developed to modulate diseased cellular networks.^22,26,27^ Accordingly, the rationale for polypharmacology (i.e. the promiscuous modulation of several molecular targets simultaneously)^26^ has progressively garnered support in both academia and industry. Understanding the plurifunctional underpinnings of miRNA and its relationship to global SUMOylation, we sought to develop a screening system capable of identifying MEs capable of influencing post-ischemic clinical outcomes.

Acknowledging that luciferase-dependent assay interference in qHTS cannot be entirely eliminated, it is nonetheless possible to significantly reduce the probability of its occurrence through the rational engineering of one’s reporter system and via the selection of appropriate orthogonal assays to confirm compound activity. Both of the aforementioned represent the best means of identifying artifactual activity early in drug discovery processes.^28^ Further, many of the compounds within screening libraries directly perturb the activity of luciferase reporters, thus skewing data interpretation and complicating candidate selection.^28–31^ In order to facilitate the construction of a functional cell-based qHTS assay capable of accurately identifying inhibitors of our miRNAs of interest, we constructed stable dual firefly-Renilla luciferase reporter lines, thereby reducing the number of downstream manipulations and enhancing concurrently the overall reproducibility of the assay.

To minimize interference and increase our confidence in hits during screening, we designed a construct that contains two different reporters. Of note, firefly luciferase and Renilla luciferase are not homologous and therefore have unrelated bioluminescent properties; firefly luciferase uses d-luciferin as its substrate, while Renilla luciferase makes use of coelenterazine as its substrate.^32^ Our assay rules out false-positives due to compound toxicity, which can occur in an assay based on a decrease in reporter signal. Moreover, since compounds identified using this proprietary screening approach may still have off-target effects, they need to be validated using orthogonal assays following the primary assay to differentiate between compounds that generate false positives verse those compounds that are specifically active against the target. As such, we confirmed our screening with a series of western blots and in so doing demonstrated the overall reliability of our assay.

The final strength of this novel dual reporter assay system is its downstream utility in exploring the drugability of other miRNAs of clinical interest in a qHTS manner (i.e. via the insertion of the appropriate target sequences into the pmirGLO/psiCHECK1 firefly and Renilla 3′ UTRs) expanding upon the elegant technique originally put forth by Connelly et al.^33^

Of the 21 compounds that definitively emerged from the screen and the following orthogonal assay, we noted that five were HDAC inhibitors: romidepsin, panobinostat, entinostat, belinostat, and pracinostat. HDAC inhibitors are a class of drugs that increase the acetylation of histone and non-histone proteins to activate transcription, enhance gene expression, and modify the function of target proteins.^34^ HDAC inhibitors have been shown in a myriad of basic and preclinical studies to provide vigorous protection against excitotoxicity, oxidative and endoplasmic reticulum stress, apoptosis, inflammation, and blood brain barrier breakdown, all of which are core components of the pathobiology caused by an acute ischemic brain insult (reviewed in Fessler et al.^34^). Beyond the suppression of post-stroke injury, HDAC inhibitors have been shown capable of augmenting recovery through the promotion of angiogenesis, neurogenesis, and stem cell migration, thereby dramatically increasing both functional and behavioral recovery after experimental cerebral ischemia.^34^ Intriguingly, work has emerged to link HDAC inhibitors to the regulation of miRNA in ischemia^35^ and cancer models.^36,37^ Of particular interest, Blakeslee et al. have provided a direct link between HDAC inhibition and SUMOylation in both cardiomyocytes and fibroblasts that may help explain the beneficial effects of HDAC inhibitors in preclinical models of heart failure.^38^ Of note, the protective influences of the HDAC inhibitors have a foil in the class’ baseline toxicity, and it has therefore proven difficult to optimize the concentrations that may ultimately achieve maximal protection from OGD/ROG.^39^ Strategies including pulsed treatment may be employed to mitigate such toxicity moving forward.^40^

The compound that had the most pronounced effect in both cell lines (SHSY5Y and cortical neurons) was AHPN. Disruption of retinoid signaling has been linked to the pathological hallmarks of a number of neuroinflammatory/neurodegenerative diseases that share components of ischemic pathobiology.^41,42^ As such, both endogenous and synthetic retinoids (AHPN) have been examined for their ability to modulate inflammation via interactions with both macrophages and microglia.^41,43,44^ Previous reports have documented AHPN’s ability to act as a chemotherapeutic via an inhibition of cellular proliferation and/or induction of apoptotic cell death.^45,46^ Despite this, the work by Farso et al. has effectively demonstrated AHPN’s ability, at low concentrations, to attenuate microglial activation-associated responses without triggering cell death.^41^ Such work highlights the critical nature of AHPN dose to related biological outcomes and suggests that more work will be needed to fully elucidate this molecule’s protective capacity. It is again prudent to note that a number of the other compounds which proved capable of providing protection against the injurious effects of OGD/ROG in the primary cortical neurons have been linked previously with neuroprotection after ischemia; accordingly, orotic acid, diazoxide and telmisartan have all been shown capable of modulating relevant components of ischemic pathobiology.^47–49^

Of interest, the response of both cell types (SHSY5Y and cortical neurons) to the drugs differed and the level of protection induced did not directly correlate with the level of global SUMOylation, as may have been expected. We therefore propose that the SUMOylation of specific target sets may be a critical component of the protection afforded against ischemia in addition to global SUMOylation levels as evidenced by the recent work of Yang et al.^50^ Such differences may ultimately be exploited to further elucidate the dynamics controlling both global SUMOylation and its influence on neuroprotection during OGD/ROG.

Understanding both the burden of disease caused by ischemic stroke and our concurrent lack of cell protective therapeutic options, we sought to engineer and optimize a novel mechanism to identify molecules capable of inducing global SUMOylation via the inhibition of miRNA. Such work has considerable potential, and it is our hope that it will lead to advanced therapies that may be used to significantly reduce morbidity/mortality following ischemic brain injury, thereby improving quality of life for both patients and their families.

Future work will seek to optimize doses (of single drugs and combinations) both *in vitro* and ultimately *in vivo* to further explore the potential clinical translation of our findings.

## Supplementary Material

Supplementary material

## References

[1] Go AS Mozaffarian D Roger VL Heart disease and stroke statistics–2013 Update: A report from the American Heart Association. Circulation 2013; 127: E6–E245. 23239837 10.1161/CIR.0b013e31828124ad PMC5408511

[2] Lee YJ Hallenbeck JM SUMO and ischemic tolerance. Neuromol Med 2013; 15: 771–781. 10.1007/s12017-013-8239-9 23775726

[3] Gareau JR Lima CD The SUMO pathway: Emerging mechanisms that shape specificity, conjugation and recognition. Nat Rev Mol Cell Bio 2010; 11: 861–871. 21102611 10.1038/nrm3011 PMC3079294

[4] Mukhopadhyay D Dasso M Modification in reverse: the SUMO proteases. Trends Biochem Sci 2007; 32: 286–295. 17499995 10.1016/j.tibs.2007.05.002

[5] Flotho A Melchior F Sumoylation: A regulatory protein modification in health and disease. Annu Rev Biochem 2013; 82: 357–385. 23746258 10.1146/annurev-biochem-061909-093311

[6] Lee Y Miyake S Wakita H Protein SUMOylation is massively increased in hibernation torpor and is critical for the cytoprotection provided by ischemic preconditioning and hypothermia in SHSY5Y cells. J Cerebr Blood F Metab 2007; 27: 950–962. 10.1038/sj.jcbfm.9600395 PMC2396349 16955077

[7] Frerichs KU Kennedy C Sokoloff L Local cerebral blood flow during hibernation, a model of natural tolerance to “cerebral ischemia". J Cerebral Blood Flow Metab: official journal of the International Society of Cerebral Blood Flow and Metabolism 1994; 14: 193–205. 10.1038/jcbfm.1994.26 8113316

[8] Carey HV Andrews MT Martin SL Mammalian hibernation: Cellular and molecular responses to depressed metabolism and low temperature. Physiol Rev 2003; 83: 1153–1181. 14506303 10.1152/physrev.00008.2003

[9] Lee YJ Castri P Bembry J SUMOylation participates in induction of ischemic tolerance. J Neurochem 2009; 109: 257–267. 19200349 10.1111/j.1471-4159.2009.05957.x PMC2692380

[10] Lee YJ Mou Y Maric D Elevated global SUMOylation in Ubc9 transgenic mice protects their brains against focal cerebral ischemic damage. Plos ONE 2011; 6(10): e25852. 22016779 10.1371/journal.pone.0025852 PMC3189225

[11] Lee Y-j Mou Y Klimanis D Global SUMOylation is a molecular mechanism underlying hypothermia-induced ischemic tolerance. Front Cell Neurosci 2014; 8: 416. 25538566 10.3389/fncel.2014.00416 PMC4255597

[12] Lee YJ Johnson KR Hallenbeck JM Global protein conjugation by ubiquitin-like-modifiers during ischemic stress is regulated by microRNAs and confers robust tolerance to ischemia. Plos ONE 2012; 7: e47787. 23094087 10.1371/journal.pone.0047787 PMC3475703

[13] Dehdashti SJ Zheng W Gever JR A high-throughput screening assay for determining cellular levels of total tau protein. Curr Alzheimer Res 2013; 10: 679–687. 23905996 10.2174/15672050113109990143 PMC4010324

[14] Griner LAM Guha R Shinn P High-throughput combinatorial screening identifies drugs that cooperate with ibrutinib to kill activated B-cell-like diffuse large B-cell lymphoma cells. Proc Natl Acad Sci U S A 2014; 111: 2349–2354. 24469833 10.1073/pnas.1311846111 PMC3926026

[15] Huang R Southall N Wang Y The NCGC pharmaceutical collection: a comprehensive resource of clinically approved drugs enabling repurposing and chemical genomics. Sci Transl Med 2011; 3: 80ps16. 10.1126/scitranslmed.3001862 PMC3098042 21525397

[16] Andres RH Horie N Slikker W Human neural stem cells enhance structural plasticity and axonal transport in the ischaemic brain. Brain: A journal of neurology 2011; 134: 1777–1789. 21616972 10.1093/brain/awr094 PMC3102243

[17] Tambalo S Peruzzotti-Jametti L Rigolio R Functional magnetic resonance imaging of rats with experimental autoimmune encephalomyelitis reveals brain cortex remodeling. J Neurosci 2015; 35: 10088–10100. 26157006 10.1523/JNEUROSCI.0540-15.2015 PMC4495237

[18] Southall NT, Jadhav A, Huang R, et al. Enabling the large-scale analysis of quantitative high-throughput screening data. In: *Handbook of Drug Screening*, pp.442--464.

[19] Buddle M Eberhardt E Ciminello LH Microtubule-associated protein 2 (MAP2) associates with the NMDA receptor and is spatially redistributed within rat hippocampal neurons after oxygen-glucose deprivation. Brain Res 2003; 978: 38–50. 12834896 10.1016/s0006-8993(03)02758-6

[20] Lei Z Ruan Y Yang AN NMDA receptor mediated dendritic plasticity in cortical cultures after oxygen-glucose deprivation. Neurosci Lett 2006; 407: 224–229. 16979291 10.1016/j.neulet.2006.06.019

[21] Lu J Clark AG Impact of microRNA regulation on variation in human gene expression. Genome research 2012; 22: 1243–1254. 22456605 10.1101/gr.132514.111 PMC3396366

[22] Csermely P Korcsmaros T Kiss HJM Structure and dynamics of molecular networks: A novel paradigm of drug discovery A comprehensive review. Pharmacol Therapeut 2013; 138: 333–408. 10.1016/j.pharmthera.2013.01.016 PMC3647006 23384594

[23] Lewis BP Shih IH Jones-Rhoades MW Prediction of mammalian microRNA targets. Cell 2003; 115: 787–798. 14697198 10.1016/s0092-8674(03)01018-3

[24] Guo HL Ingolia NT Weissman JS Mammalian microRNAs predominantly act to decrease target mRNA levels. Nature 2010; 466: 835–U66. 20703300 10.1038/nature09267 PMC2990499

[25] Doench JG Sharp PA Specificity of microRNA target selection in translational repression. Genes Develop 2004; 18: 504–511. 15014042 10.1101/gad.1184404 PMC374233

[26] Hopkins AL Mason JS Overington JP Can we rationally design promiscuous drugs? Curr Opin Struct Biol 2006; 16: 127–136. 16442279 10.1016/j.sbi.2006.01.013

[27] Nacher JC Schwartz JM A global view of drug-therapy interactions. BMC Pharmacol 2008; 8: 5. 18318892 10.1186/1471-2210-8-5 PMC2294115

[28] Thorne N Auld DS Inglese J Apparent activity in high-throughput screening: origins of compound-dependent assay interference. Curr Opin Chem Biol 2010; 14: 315–324. 20417149 10.1016/j.cbpa.2010.03.020 PMC2878863

[29] Auld DS Southall NT Jadhav A Characterization of chemical libraries for luciferase inhibitory activity. J Med Chem 2008; 51: 2372–2386. 18363348 10.1021/jm701302v

[30] Thompson JF Hayes LS Lloyd DB Modulation of firefly luciferase stability and impact on studies of gene regulation. Gene 1991; 103: 171–177. 1889744 10.1016/0378-1119(91)90270-l

[31] Auld DS Thorne N Nguyen DT A specific mechanism for nonspecific activation in reporter-gene assays. ACS Chem Biol 2008; 3: 463–470. 18590332 10.1021/cb8000793 PMC2729322

[32] Loening AM Fenn TD Gambhir SS Crystal structures of the luciferase and green fluorescent protein from Renilla reniformis. J Mol Biol 2007; 374: 1017–1028. 17980388 10.1016/j.jmb.2007.09.078 PMC2700051

[33] Connelly CM Thomas M Deiters A High-throughput luciferase reporter assay for small-molecule inhibitors of MicroRNA function. J Biomol Screen 2012; 17: 822–828. 22412086 10.1177/1087057112439606 PMC3758890

[34] Fessler EB Chibane FL Wang ZF Potential roles of HDAC inhibitors in mitigating ischemia-induced brain damage and facilitating endogenous regeneration and recovery. Curr Pharmaceut Des 2013; 19: 5105–5120. 10.2174/1381612811319280009 PMC6322545 23448466

[35] Hunsberger JG Fessler EB Wang Z Post-insult valproic acid-regulated microRNAs: potential targets for cerebral ischemia. Am J Transl Res 2012; 4: 316–332. 22937209 PMC3426385

[36] Lodrini M Oehme I Schroeder C MYCN and HDAC2 cooperate to repress miR-183 signaling in neuroblastoma. Nucleic Acid Res 2013; 41: 6018–6033. 23625969 10.1093/nar/gkt346 PMC3695529

[37] Scott GK Mattie MD Berger CE Rapid alteration of microRNA levels by histone deacetylase inhibition. Cancer Res 2006; 66: 1277–1281. 16452179 10.1158/0008-5472.CAN-05-3632

[38] Blakeslee WW, Wysoczynski CL, Fritz KS, et al. Class I HDAC inhibition stimulates cardiac protein SUMOylation through a post-translational mechanism. *Cell Signal* 2014; 26: 2912--2920. 10.1016/j.cellsig.2014.09.005 PMC4252807 25220405

[39] Langley B D'Annibale MA Suh K Pulse inhibition of histone deacetylases induces complete resistance to oxidative death in cortical neurons without toxicity and reveals a role for cytoplasmic p21(waf1/cip1) in cell cycle-independent neuroprotection. J Neurosci: the official journal of the Society for Neuroscience 2008; 28: 163–176. 10.1523/JNEUROSCI.3200-07.2008 PMC2577229 18171934

[40] Langley B Brochier C Rivieccio MA Targeting histone deacetylases as a multifaceted approach to treat the diverse outcomes of stroke. Stroke; a journal of cerebral circulation 2009; 40: 2899–905. 10.1161/STROKEAHA.108.540229 19478231

[41] Farso MC Krantic S Rubio M The retinoid, 6-[3-adamantyl-4-hydroxyphenyl]-2-napthalene carboxylic acid, controls proliferative, morphological, and inflammatory responses involved in microglial activation without cytotoxic effects. Neuroscience 2011; 192: 172–184. 21749910 10.1016/j.neuroscience.2011.06.053

[42] Maden M Retinoic acid in the development, regeneration and maintenance of the nervous system. Nat Rev Neurosci 2007; 8: 755–765. 17882253 10.1038/nrn2212

[43] Diab A Hussain RZ Lovett-Racke AE Ligands for the peroxisome proliferator-activated receptor-gamma and the retinoid X receptor exert additive anti-inflammatory effects on experimental autoimmune encephalomyelitis. J Neuroimmunol 2004; 148: 116–126. 14975592 10.1016/j.jneuroim.2003.11.010

[44] Xu J Drew PD 9-Cis-retinoic acid suppresses inflammatory responses of microglia and astrocytes. J Neuroimmunol 2006; 171: 135–144. 16303184 10.1016/j.jneuroim.2005.10.004 PMC2825699

[45] Li Y Lin BZ Agadir A Molecular determinants of AHPN (CD437)-induced growth arrest and apoptosis in human lung cancer cell lines. Mol Cell Biol 1998; 18: 4719–4731. 9671482 10.1128/mcb.18.8.4719 PMC109058

[46] Zhao XS Spanjaard RA The apoptotic action of the retinoid CD437/AHPN: Diverse effects, common basis. J Biomed Sci 2003; 10: 44–49. 12566985 10.1007/BF02255996

[47] Akiho H Iwai A Katoh-Sudoh M Neuroprotective effect of YM-39558, orotic acid ethylester, in gerbil forebrain ischemia. Japan J Pharmacol 1998; 76: 441–444. 9623724 10.1254/jjp.76.441

[48] Dutta S Rutkai I Katakam PV The mechanistic target of rapamycin (mTOR) pathway and S6 Kinase mediate diazoxide preconditioning in primary rat cortical neurons. J Neurochem 2015; 134: 845–856. 26016889 10.1111/jnc.13181 PMC5667543

[49] Wang J Pang T Hafko R Telmisartan ameliorates glutamate-induced neurotoxicity: roles of AT(1) receptor blockade and PPARgamma activation. Neuropharmacology 2014; 79: 249–261. 24316465 10.1016/j.neuropharm.2013.11.022 PMC3950310

[50] Yang W Sheng H Thompson JW Small ubiquitin-like modifier 3-modified proteome regulated by brain ischemia in novel small ubiquitin-like modifier transgenic mice: putative protective proteins/pathways. Stroke; a journal of cerebral circulation 2014; 45: 1115–1122. 10.1161/STROKEAHA.113.004315 PMC3966925 24569813

