# Supplementary material for: A novel quantitative high-throughput screen identifies drugs that both activate SUMO conjugation via the inhibition of microRNAs 182 and 183 and facilitate neuroprotection in a model of oxygen and glucose deprivation
Source: J Cereb Blood Flow Metab. 2015 Oct 23;36(2):426–41. doi: 10.1177/0271678X15609939 (PMC4759677; doi:10.1177/0271678X15609939)
Supplement: Supplementary material [file Supplementary_Material_939.pdf]

For **pmirGLO** (between PmeI and XbaI, with NotI as an internal site)

PmeI NotI miR-182/183 target sequence XbaI

miR-182 sense: 5'-**AAAC**TAGCGGCCGCTAGTAGTGTGAGTTCTACCATTGCCAAA**T**-3'
miR-183 sense: 5'-**AAAC**TAGCGGCCGCTAGTAGTGAATTCTACCAGTGCCATA**T**-3'

XbaI miR-182/183 target sequence NotI PmeI

miR-182 antisense: 5'-**CTAGA**TTTGGCAATGGTAGAACTCACACTACTAGCGGCCGCTA**GTTT**-3'
miR-183 antisense: 5'-**CTAGA**TATGGCACTGGTAGAATTCACTACTAGCGGCCGCTA**GTTT**-3'

For **psiCHECK1** (between SgfI and PmeI, with SacI as an internal site)

SgfI SacI miR-182/183 target sequence PmeI

miR-182 sense: 5'-**CGC**AGTAGAGCTCTAGTA**AGTGTGAGTTCTACCATTGCCAAAGTTT**-3'
miR-183 sense: 5'-**CGC**AGTAGAGCTCTAGTAAGTGAATTCTACCAGTGCCATA**GTTT**-3'

PmeI miR-182 /183 target sequence SacI SgfI

miR-182 antisense: 5'- **AAACTTTGGCAATGGTAGAACTCACACT**TACTAGAGCTCTACT**GCGAT** -3'
miR-183 antisense : 5'- **AAAC**TATGGCACTGGTAGAATTCACTTACTAGAGCTCTACT**GCGAT**-3'

Supplemental Fig. 2

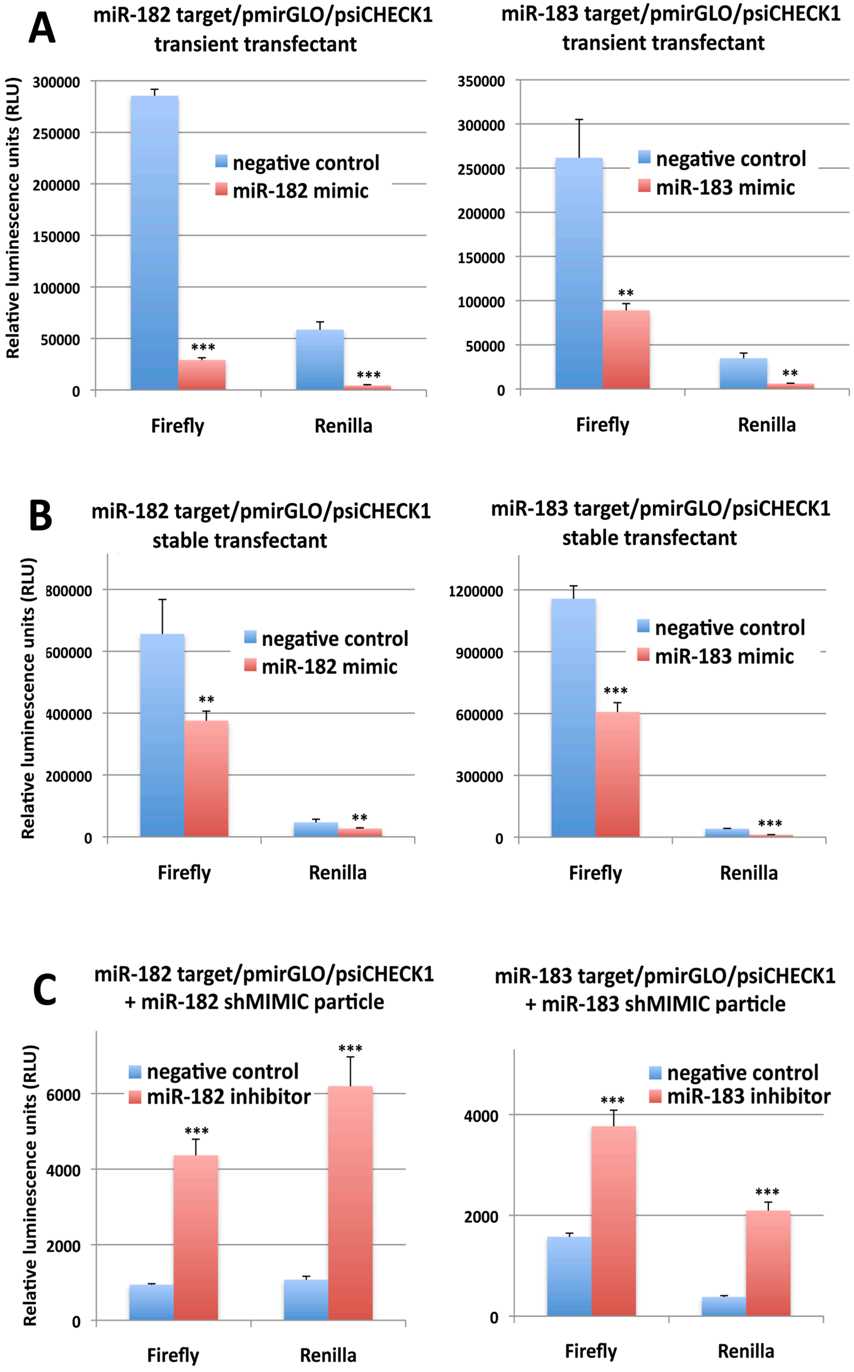

Supplemental Fig. 3

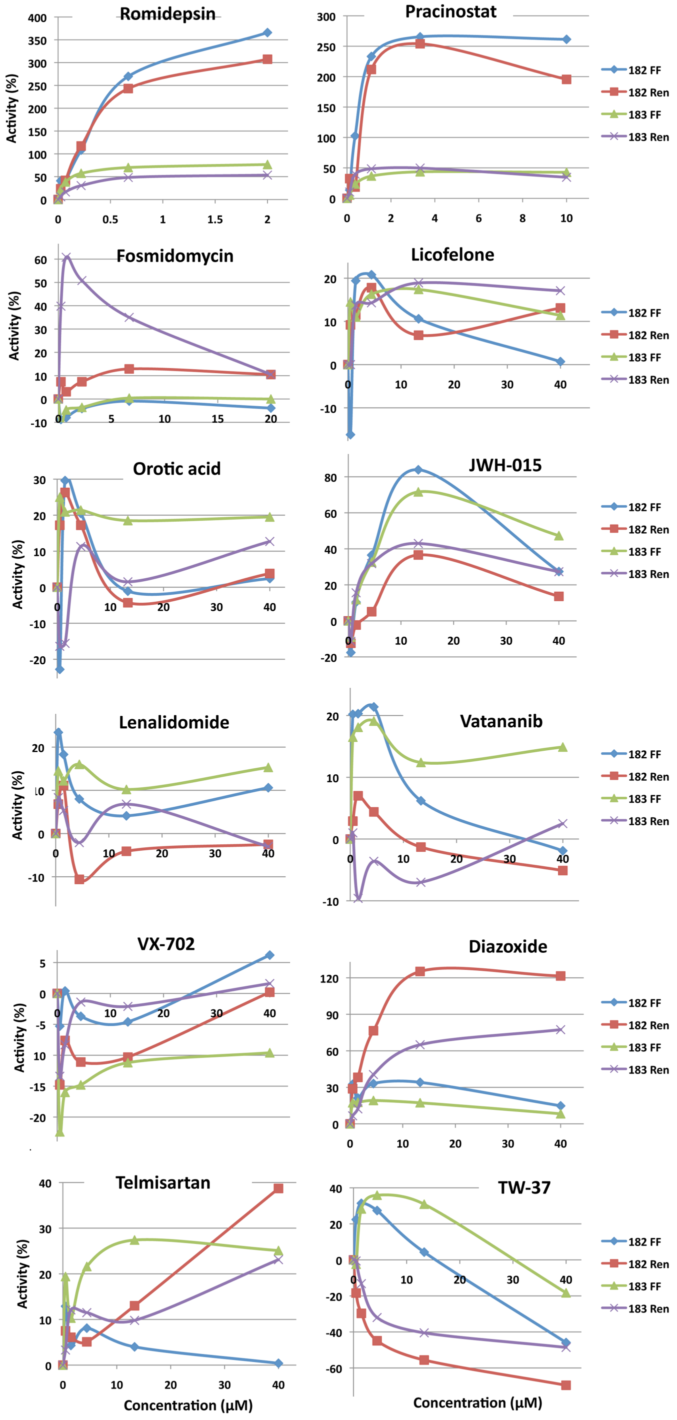

A

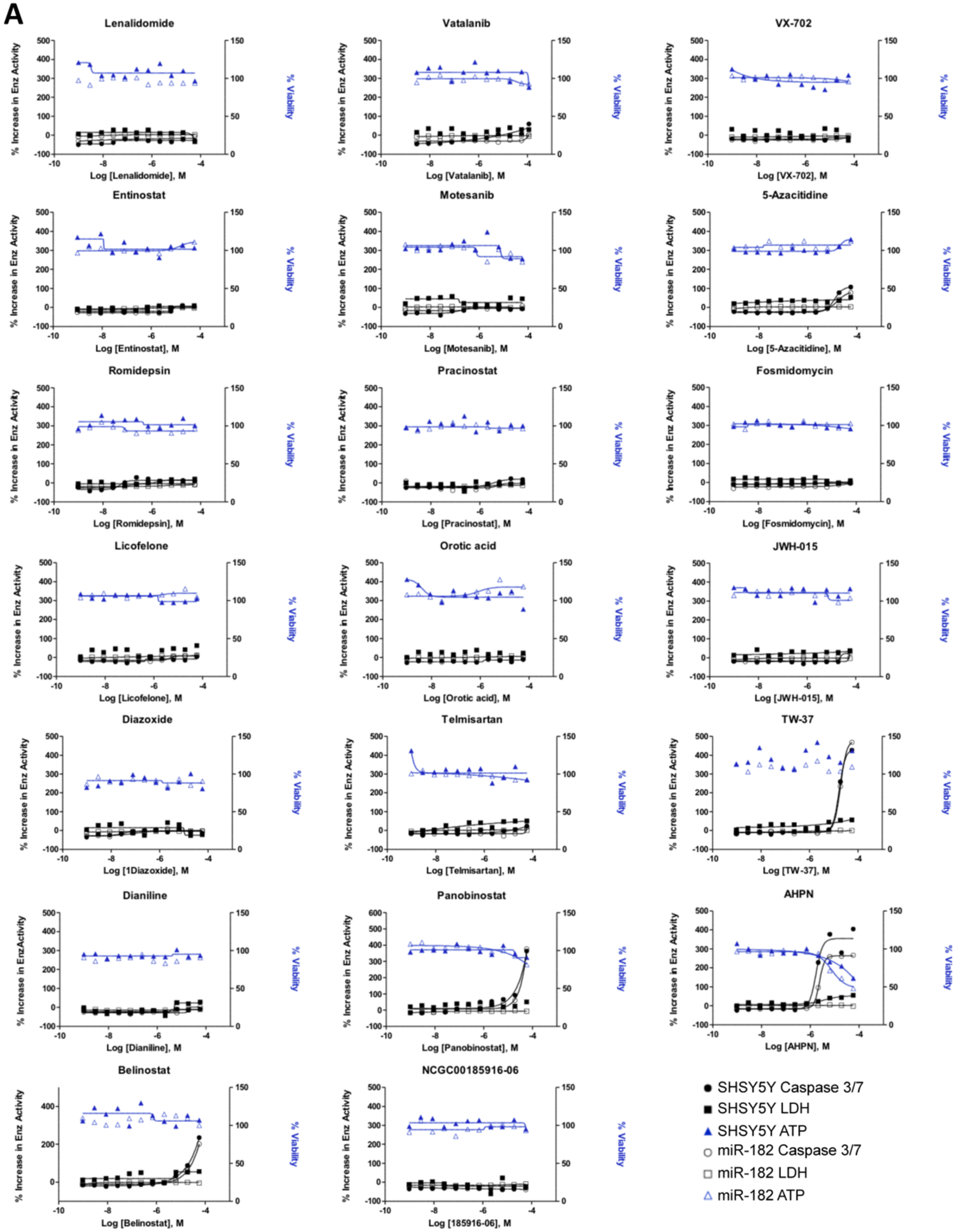

B

Compound	Name	Cytotoxicity?	Western Low Dose (µM)	Western High Dose (µM)
15380-09	Diazoxide	N	5	50
165833-04	Entinostat	N	2	10
249684-01	VX-702; KVK-702	N	10	50
264107-01	Fosmidomycin	N	0.2	2
181350-02	Vatalanib	Caspase 3/7	2	10
167491-03	Lenalidomide	N	5	50
95150-06	Telmisartan	N	10	50
92284-02	AHPN	Caspase 3/7; ATP	5	20
263205-01	Motesanib	N	10	50
90851-08	5-Azacitidine	Caspase 3/7	1	10
250374-01	Licofelone	N	2	20
91357-01	Orotic acid	N	2	10
263155-01	Belinostat	Caspase 3/7	2	10
15575-04	JWH-015	N	10	50
263208-01	TW-37	Caspase 3/7	2	20
263117-01	Panobinostat	Caspase 3/7	0.2	2
160373-01	Dianiline	N	10	50
185916-06	NCGC00185916	N	5	20
263136-03	Pracinostat	N	1	5
263220-01	Romidepsin	N	0.1	1

Supplemental Table 1

	Molecular Entity	OGD (µM)
1	Romidepsin	0.1
2	Panobinostat	0.05
3	Entinostat	2
4	Belinostat	2
5	Pracinostat	1
6	Fosmidomycin	2
7	NCGC00185916	5
8	Licofelone	2
9	Motesanib	5
10	Orotic acid	5
11	JWH-015	10
12	5-Azacitidine	1
13	AHPN	1
14	Lenalidomide	10
15	Vatalanib	5
16	VX-702;KVK-702	10
17	Dianiline	5
18	Diazoxide	5
19	Telmisartan	10
20	JQ1	0.5
21	TW-37	2

Supplemental Table 2

Compound	Number of IPA[1] associated Genes	Number of IPA[1] stroke-associated[2] Genes	Chi-square[3] yates corrected P	IPA[1] stroke-associated Genes
Romidepsin	70	7	<0.0001	HDAC9,INSR,ITGAV,MMP9,PTGS2,SLC1A2,TP53
Panobinostat	33	1	0.0166	PTGS2
Entinostat	84	3	<0.0001	AIFM1,HDAC9,MMP9
Belinostat	13	1	<0.0001	HDAC9
Licofelone	2	2	<0.0001	PTGS1,PTGS2
Motesanib	5	0	0.977	NA
Orotic acid	15	0	0.931	NA
JWH-015	14	2	<0.0001	NOS3,RHOA
5-Azacitidine	124	2	0.1712	NOS3,PTGS2
AHPN	223	4	0.1554	CCL5,FAS,S100P,TP53
Lenalidomide	136	2	0.2641	GABRB2,TP53
Vatalanib	14	1	<0.0001	MMP9
Diazoxide	47	4	<0.0001	ADIPOQ,AIFM1,GJA1,INS
Telmisartan	65	6	<0.0001	ADIPOQ,AGTR1,MMP9,NOS3,PTGS2,SLC6A3

[1] IPA: Ingenuity Pathway Analysis (www.ingenuity.com)

[2] Total number of IPA[1] stroke-associated genes = 143

[3] Total number of genes in genome = 30k

Supplemental Figure Legends

- **Supplemental 1. Oligonucleotide sequences.** Oligonucleotide pairs were engineered to contain the miR-182 (or miR-183) target sequence with restriction sites (PmeI and XbaI for pmirGLO, and SgfI and PmeI for psiCHECK-1, and an internal restriction site (NotI for pmirGLO and SacI for psiCHECK-1) for clone confirmation.
- **Supplemental 2. Confirmation of final constructs and/or stable transfectants for the reporter assays. A**: SHSY5Y parent cells were transiently co-transfected with the final constructs (miR-182 target or miR-183 target) and either a miR negative control or miR-182 (or miR-183) mimic. The firefly and Renilla luciferase activities were measured after 24 h of incubation. **B**: Stable SHSY5Y transfectants that contained the final constructs (either miR-182 target or miR-183 target in pmirGLO/psiCHECK-1) were transfected with either a miR negative control or miR-182 (or miR-183) mimic. The firefly and Renilla luciferase activities were measured after 24 h of incubation. **C**: Stable SHSY5Y transfectants that contained the final constructs (either miR-182 target or miR-183 target in pmirGLO/psiCHECK-1) plus lentiviral particles containing miR-182 (or miR-183) shMIMIC were transfected with either miR-182 (or miR-183) specific inhibitors or non-specific miRNA negative controls. The firefly and Renilla luciferase activities were measured after 24 h of incubation. Of note all assays were conducted in quintuplicate utilizing a 96-well format.
- **Supplemental 3**. **Dose response of remaining MEs identified by qHTS.**
- **Supplemental 4**. **Cytotoxicity assays**. **A**: SHSY5Y and miR182 cells were plated at 2000 cells/well in 4uL/well and incubated O/N (16 hr) at 37C, 5% CO2. Compounds were incubated with cells for 13.5 hours and Caspase 3/7, LDH, and ATP fluorescent/luminescent assays were performed. **B**. List of the tested compounds and doses.
- **Supplemental Table 1. Concentrations of MEs utilized during OGD**
- **Supplemental Table 2. Stroke associated genes per ME**
